# Evaluation of Two Models for Human Topoisomerase I Interaction with dsDNA and Camptothecin Derivatives

**DOI:** 10.1371/journal.pone.0024314

**Published:** 2011-08-30

**Authors:** Gary S. Laco

**Affiliations:** Laboratory of Computational and Molecular Biochemistry, Lake Erie College of Osteopathic Medicine, Bradenton, Florida, United States of America; The University of Kansas Medical Center, United States of America

## Abstract

Human topoisomerase I (Top1) relaxes supercoiled DNA during cell division. Camptothecin stabilizes Top1/dsDNA covalent complexes which ultimately results in cell death, and this makes Top1 an anti-cancer target. There are two current models for how camptothecin and derivatives bind to Top1/dsDNA covalent complexes (Staker, et al., 2002, Proc Natl Acad Sci USA 99: 15387–15392; and Laco, et al., 2004, Bioorg Med Chem 12: 5225–5235). The interaction energies between bound camptothecin, and derivatives, and Top1/dsDNA in the two models were calculated. The published structure-activity-relationships for camptothecin and derivatives correlated with the interaction energies for camptothecin and derivatives in the Laco et al. model, however, this was not the case for several camptothecin derivatives in the Stacker et al. model. By defining the binding orientation of camptothecin and derivatives in the Top1/dsDNA active-site these results allow for the rational design of potentially more efficacious camptothecin derivatives.

## Introduction

Camptothecin (CPT) is a natural product that was isolated from the Chinese tree *Camptotheca acuminata* by Wall and Wani and was shown to inhibit the growth of cancer cells in cell culture [1]. Derivatives of CPT, including topotecan and irinotecan, have been approved by the Food and Drug Administration for the treatment of cancer. Several groups have shown that CPT specifically targets human topoisomerase type IB (Top1) [2], [3]. Top1 binds dsDNA and attacks the backbone scissile bond phosphate with the active-site Tyr723 to make a covalent tyrosyl-phosphate bond to the -1 scissile strand deoxyribonucleotide (-1 nucleotide). This results in a +1 deoxyribonucleoside (+1 nucleoside) with a free 5′-OH. CPT stabilizes the Top1/dsDNA covalent complex [4]. When cells replicate DNA containing CPT stabilized Top1/dsDNA covalent complexes the single-strand breaks are converted into dsDNA breaks and it is thought that this ultimately results in cell death [5]–[7]. Numerous derivatives of CPT have been designed and synthesized to increase specificity and bioavailability, reviewed in [8], [9]. However, one problem associated with the use of CPT derivatives in the treatment of breast cancer is the development of drug resistance [9]. Breast cancer resistance has been linked to the over expression of the breast cancer resistance protein, the substrates for which are planar with conjugated pi-orbitals and with an OH or amino group at the A-ring 10 position of CPT derivatives [10]. A model of CPT binding in the Top1/dsDNA active-site would allow for the rational design of CPT derivatives which are not substrates for the breast cancer resistance protein.

A key result in the Top1 field was obtained when Redinbo et al. solved the X-ray crystal structure of Top1 in covalent complex with a suicide-DNA [11]. Since Top1 has a low affinity for relaxed dsDNA, a suicide dsDNA oligonucleotide (suicide-DNA) was used to generate the Top1/suicide-DNA covalent complexes [11]. The suicide-DNA differed from native dsDNA in that the backbone scissile bond 5′ oxygen (O5′) was replaced with a sulfur. When the Top1 active-site Tyr723 attacked the suicide-DNA it generated a native tyrosyl-phosphate bond to the 3′ end of the -1 nucleotide while leaving a non-native 5′-SH on the free end of the +1 nucleoside [11], [12]. And while the 5′-SH can attack the tyrosyl-phosphate bond resulting in religation of the scissile strand DNA backbone, the equilibrium is shifted ∼7-fold towards the Top1/dsDNA covalent complex [13]. As a result, Top1 remained trapped in covalent complex with the suicide-DNA in the crystals [11]. The Redinbo et al. structure allowed for the structure-based modeling of CPT and derivatives in the Top1/suicide-DNA active site and resulted in models by Redinbo et al. [11], Kerrigan et al. [14], and Laco et al. [15], the last of which is described here as the Rotated +1 Nucleoside model.

Of these structure-based Top1/dsDNA/inhibitor models, only the orientation of CPT and derivatives in the Rotated +1 Nucleoside model were experimentally tested *in vitro* using both a Top1 Asn352Ala mutant and a dsDNA oligonucleotide assay which used cobalt to oxidize extra helical guanines [15], [16]. In the Laco et al. Rotated +1 Nucleoside model, the +1 nucleoside rotated out of the helix when Top1 was in covalent complex with supercoiled dsDNA. As a result of the rotation, the free 5′OH of the +1 nucleoside was out of range (7.7 Å) of the tyrosyl-phosphate bond preventing religation of the DNA backbone [15]. This finding supports the processive nature of Top1 in that it stays bound to supercoiled dsDNA until the dsDNA is fully relaxed [17]. After the dsDNA is fully relaxed, the +1 nucleoside is rotated back into the helix allowing the +1 nucleoside 5′OH to attack the tyrosyl-phosphate bond, religate the DNA backbone, and allow Top1 to dissociate from the fully relaxed dsDNA [15]. Rotation of the +1 nucleoside out of the helix leaves a cavity in the Top1/dsDNA active-site which can be occupied by CPT and derivatives. When CPT and derivatives bind in the Rotated +1 Nucleoside model Top1/dsDNA active-site they block the +1 nucleoside from re-entering the helix thus stabilizing the Top1/dsDNA covalent complex [15].

The Rotated +1 Nucleoside model is significantly different from the Staker et al. X-ray crystallography based models of Top1 in covalent complex with suicide-DNA and either topotecan [18] or CPT [19]. In both Staker et al. structure based models the +1 nucleoside was based-paired in the helix, and the inhibitors intercalated in between the -1 and +1 base pairs, forcing the +1 base pairs ∼3.4 Å further away from the -1 base pairs [18], [19]. As a result, the suicide-DNA +1 nucleoside 5′SH was 11.6 Å away from the tyrosyl-phosphate bond preventing religation of the DNA [18], [19]. The Staker et al. structure-based model of Top1 in covalent complex with dsDNA and bound inhibitor is described here as the “Intercalated model”. The Rotated +1 Nucleoside model and the Intercalated model were evaluated *in silico* using Discovery Studio computational chemistry software (Accelrys, San Diego, CA). This approach allowed for evaluation of the binding orientations of CPT and derivatives in the Rotated +1 Nucleoside and Intercalated models, to see which binding orientation correlated best with the reported *in vitro* structure-activity-relationships for CPT and derivatives.

## Results and Discussion

**Figure 1:**
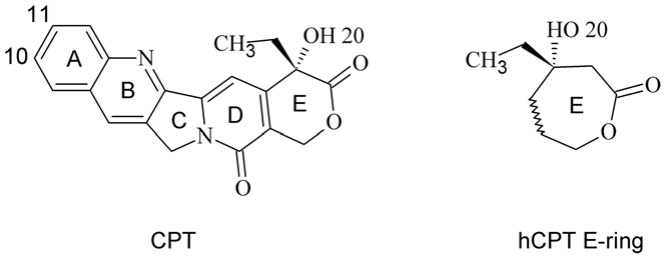
Structure of CPT and hCPT E-ring. CPT left, hCPT E-ring right; hCPT and derivatives differ from CPT in that they contain an additional E-ring carbon between the 20-OH and the adjacent carbonyl oxygen to give a seven-member E-ring.

**Figure 2:**
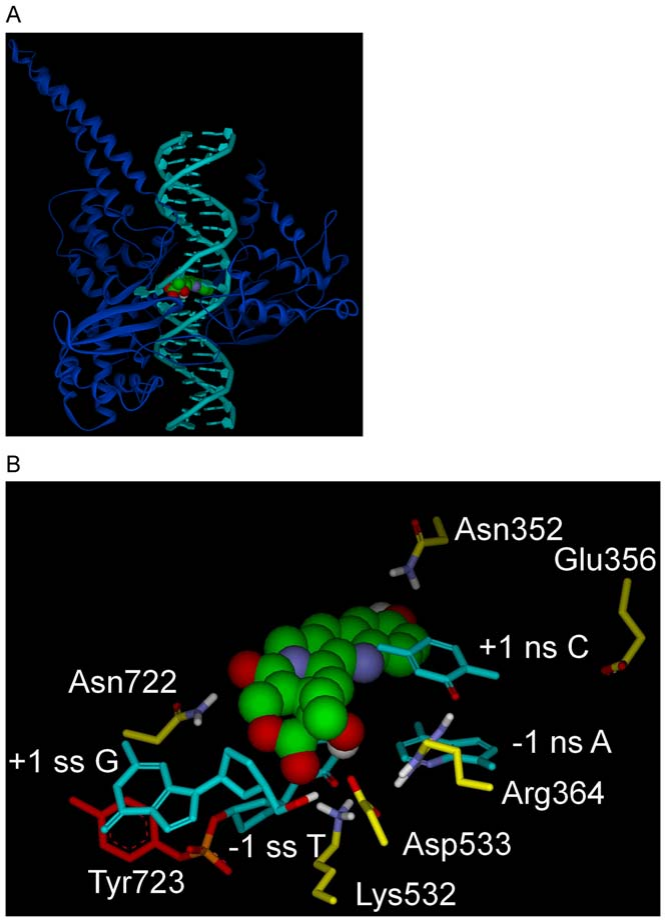
Rotated +1 Nucleoside model for Top1 interaction with dsDNA and 10-OH CPT. A) Rotated +1 Nucleoside model, Top1 shown as blue ribbon, dsDNA in teal with the rotated +1 deoxyguanosine left of 10-OH CPT (CPK rendering; carbon, green; oxygen, red; nitrogen, blue; hydrogen, white). B) Rotated +1 Nucleoside model, close up of Fig. 2A active-site. 10-OH CPT with E-ring in foreground, bound in the Top1/dsDNA active-site in which the +1 scissile strand G is rotated out of the helix to the left until trapped in a network of H-bonds/electrostatic interactions with Asp533 (center) and Arg488/Arg590 (not shown for clarity, see Fig. 4A). Selected atoms involved in H-bonds/electrostatic interactions are colored: nitrogen, blue; oxygen, red; hydrogen, white. Top1 side-chain carbons are yellow, except for Tyr723 (red) in tyrosyl-phosphate bond (phosphorus, orange) to the -1 scissile strand T. 10-OH CPT interactions: 10-OH CPT D-ring stacks over the -1 scissile strand T; 20-OH H-bonds to -1 scissile strand T carbonyl oxygen; A-ring 10-OH oxygen makes electrostatic interaction with Asn352 nitrogen (3.6 Å); E-ring carbonyl oxygen H-bonds to Lys532 nitrogen; C-ring carbonyl oxygen H-bonds to Asn722 nitrogen. Scissile strand rotated +1 G 5′OH H-bonds to Asp533. Arg364 nitrogens H-bond to +1 non-scissile strand C carbonyl oxygen and -1 non-scissile strand A nitrogen. Scissile strand, ss; non-scissile strand, ns. For flat image see Fig. 4A.

CPT and derivatives selected for this study, along with the respective *in vitro* inhibition results for each, are shown in Figure 1 and Table 1 [15], [16], [20]. There were three selection criteria for the inhibitors used in this study: 1) inhibitors had been tested in an *in vitro* Top1 dsDNA oligonucleotide assay in which the dsDNA oligonucleotide had a single high affinity Top1 cleavage site; 2) the assay measured the accumulation of Top1/dsDNA covalent complexes over time; and 3) inhibitors were tested at three or more concentrations to quantitate inhibition. As a result, topotecan was not included in the study due to the lack of published reports documenting the *in vitro* inhibition activity of topotecan using the same assay and conditions as for the inhibitors in Table 1 [21], [22]. The original Laco et al. model of Top1 in covalent complex with DNA and inhibitor [15] was based on the Redinbo et al. X-ray crystal structure of Top1 in covalent complex with suicide-DNA [11]. A reconstituted Top1 was used to generate the Redinbo et al. Top1/suicide-DNA covalent-complex structure and the following Top1 residues were not resolved in the functional domain which includes the DNA binding and enzymatic domains of Top1 (aa 201–265) [17]: residues 201–214; core residues 627–634, linker residues 635–697; and C-terminal residues 698–719 which are part of the Top1 active-site [11]. A more recent structure by Staker et al. of a Top1/suicide-DNA/topotecan ternary complex resolved all main-chain Top1 atoms in the functional domain [18]. However, in that structure the intercalated topotecan, a CPT derivative with an A-ring 9-CH_2_N(CH_3_)_2_ and 10-OH, displaced the +1 bases ∼3.4 Å further away from the cleavage-site -1 base pairs. In order to generate a structurally complete model of Top1 in covalent-complex with native dsDNA and no inhibitor the displaced DNA strands were replaced in the Stacker et al. Top1/suicide-DNA/topotecan structure [18] with the corresponding DNA strands from the Redinbo et al. Top1/suicide DNA structure [11] using Discovery Studio and the Consistent Force Field (CFF) which has parameters for protein, DNA, and small ligands (Methods). Next, the +1 nucleoside was rotated out of the helix until it was trapped in a network of H-bonds/electrostatic interactions with Arg488, Asp533, Arg590 as previously reported [15]. CPT was then docked into the active-site of the Rotated +1 Nucleoside model and modified into the indicated CPT derivatives (Figure 1 and Table 1); with 10-OH CPT shown in the Rotated +1 Nucleoside model active-site in Figure 2. The orientations of CPT and derivatives in this structurally complete Rotated +1 Nucleoside model were similar to the Laco et al. model [15] that was based on the incomplete Top1/suicide-DNA structure by Redinbo et al. [11]. In addition, the suicide-DNA backbone sulfur, that is found as a free 5′SH on the +1 nucleoside, was changed to the native oxygen [11], [18], [19]. Explicit waters were added to the Rotated +1 Nucleoside model presented here followed by minimization, however, individual waters did not make any bridging H-bonds/electrostatic interactions between 10-OH CPT and the Top1/dsDNA active-site (data not shown). This result is consistent with previously published results on the Rotated +1 Nucleoside model in which individual explicit waters did not make bridging H-bond interactions between ligands and the Top1/dsDNA active-site [15]. As a result, all Rotated +1 Nucleoside model Top1/dsDNA/inhibitor complexes were minimized with an implicit solvent model (Methods).

**Table 1:** Relative *in vitro* inhibition of Top1 by CPT, hCPT, and derivatives.

Ligand	Position			Inhibition
	10	11	20	
CPT	H	H	OH	0.9*
hCPT	H	H	OH	1.0*
± 20-deoxy CPT	H	H	*H*	0.03**
± 20-Cl CPT	H	H	*Cl*	0.29**
10-OH CPT	*OH*	H	OH	3.5*
10,11-diF hCPT	*F*	*F*	OH	12.0*

Modified A-ring ring positions (10, 11) and E-ring position (20) are indicated for CPT/hCPT derivatives; modifications shown in italics (see Fig. 1 for CPT and hCPT structures).

*As reported by Laco et al. [15], [16].

**As reported by Wang et al. for racemic CPT derivatives [20]. All Top1 inhibition assays were performed using an end-labeled dsDNA oligonucleotide.

**Figure 3:**
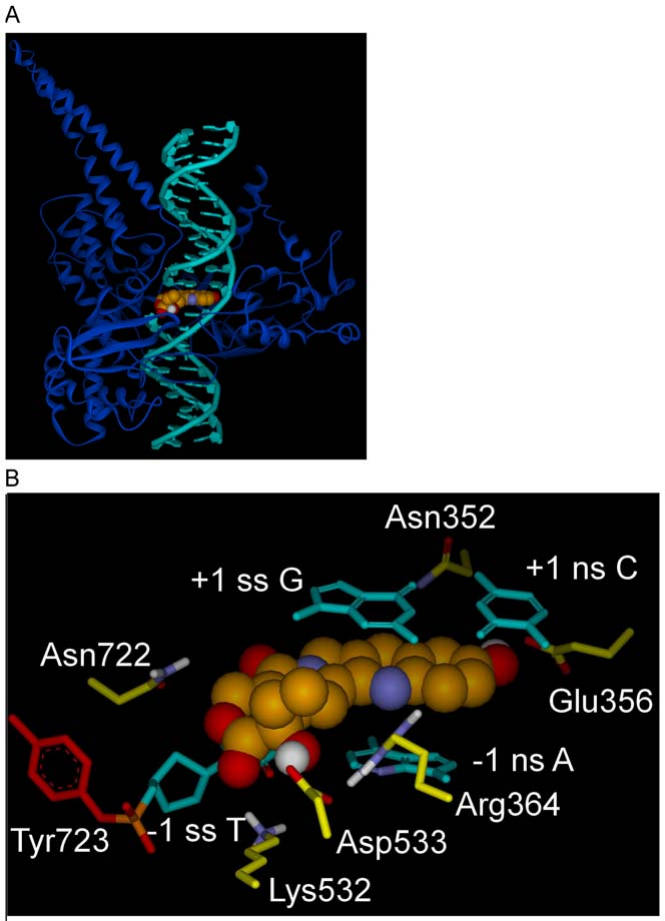
Intercalated model for Top1 interaction with dsDNA and 10-OH CPT. A) Intercalated model; Top1 shown as blue ribbon, dsDNA in teal, 10-OH CPT center (CPK rendering; carbon, bronze; oxygen, red; nitrogen, blue; hydrogen, white). B) Intercalated model, close up of Fig. 3A active-site. 10-OH CPT (carbons bronze) with E-ring in foreground intercalated between the -1 and +1 base pairs in the Top1/dsDNA active-site. 10-OH CPT interactions: 10-OH CPT stacks in between the +1 and -1 base pairs; A-ring 10-OH H-bonds to Glu356 oxygen; E-ring 20-OH H-bonds to Asp533 oxygen; D-ring carbonyl oxygen makes an electrostatic interaction with Asn722 (4.1 Å). Arg364 H-bonds with the -1 non-scissile strand T, Lys 532 H-bonds to the -1 scissile strand T carbonyl oxygen. Top1 active-site Tyr723 (red, left) is shown making a tyrosyl-phosphate bond to the -1 scissile strand T. Scissile strand, ss; non-scissile strand, ns. For flat image see Fig. 4B.

The Staker et al. structure based Intercalated model of Top1 in covalent complex with suicide-DNA and topotecan was modified [18], topotecan was converted into CPT and derivatives including 10-OH CPT, and the suicide-DNA backbone sulfur was changed to the native oxygen (Fig. 1 and 3, Table 1, Methods). In the Staker et al. structure of topotecan in ternary complex with Top1 and suicide-DNA two crystallographic waters were found to make bridging H-bonds between the topotecan carboxylate and Top1 active-site residues [18], however, carboxylate forms of CPT derivatives have been shown to be inactive [15]. One additional water was found to be within 2.9 Å of the topotecan A-ring 10-OH, but there were no Top1/dsDNA H-bond donor/acceptor residues within 4.4 Å of that water [18]. Here the Intercalated model was initially solvated with explicit waters followed by minimization, and a water was found close to the 10-OH of 10-OH CPT, however, the only other H-bond donors/acceptors within 4.4 Å were other waters (data not shown). Since no individual waters were found to make bridging H-bond/electrostatic interactions between the inhibitor and the Top1/dsDNA active-site, all Intercalated model Top1/dsDNA/inhibitor complexes were minimized with an implicit solvent model (Methods). 10-OH CPT is shown in the Intercalated model Top1/dsDNA active-site in Figure 3. The Top1/suicide-DNA/topotecan X-ray crystallography based model was used here because Top1 in the Top1/suicide-DNA/CPT model was not structurally complete [19].

The inclusion of explicit water molecules during an interaction energy calculation between an inhibitor and Top1/dsDNA would artificially make the electrostatic component of the interaction energy score more negative (i.e., stronger) when individual waters do not mediate significant bridging H-bonds/electrostatic interactions between the inhibitor and the Top1/dsDNA active-site.

### Similarities and differences between Rotated +1 Nucleoside and Intercalated Models

While the orientation of 10-OH CPT and DNA in the two models is overall strikingly different, there are similarities and they are described first. Top1 active-site residues Arg364, Lys532, and Arg533 are close to the E-ring in both models, while Asn722 faces the D-ring carbonyl oxygen in both models. In addition, the -1 scissile strand thymine base stacks under the D-ring in both models (Fig. 2 and 3).

**Figure 4:**
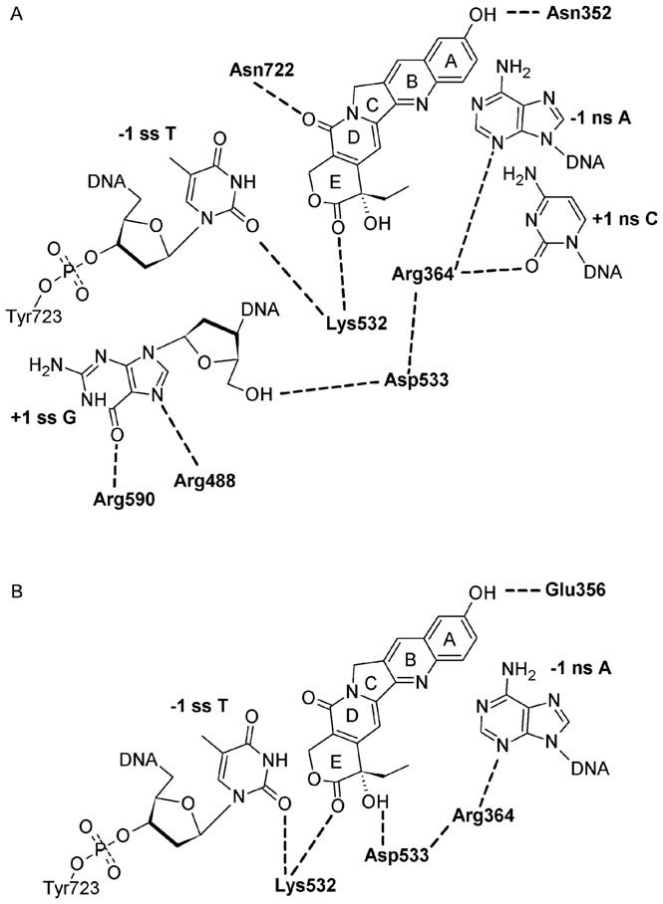
Flattened images of two models for Top1 interaction with dsDNA and 10-OH CPT. A) Flat image of Rotated +1 Nucleoside model from Fig. 2, H-bonds and electrostatic interactions within 3.6 Å are indicated with dashed lines. B) Flat image of Intercalated model from Fig. 3, H-bonds and electrostatic interactions within 3.6 Å are indicated with dashed lines.

In terms of the differences between the two models: 1) In the Rotated +1 Nucleoside model the A-ring 10-OH makes an electrostatic interaction with Asn352 (Fig. 2B and 4A), while in the Intercalated model the A-ring 10-OH H-bonds to Glu356 (Fig. 3B and 4B); 2) In the Rotated +1 Nucleoside model 10-OH CPT only stacks with the -1 scissile strand T (Fig. 2), while in the Intercalated model 10-OH CPT stacks with the -1 and +1 base pairs (Fig. 3). And while Asn430, Pro431, Leu721, and Lys751 are within 4–7.7 Å of 10-OH CPT in the Rotated +1 Nucleoside model, those residues are 1–4 Å further away from 10-OH CPT in the Intercalated model (data not shown). By determining the orientation of CPT and derivatives in the Top1 active-site, CPT derivates could be designed to make additional H-bonds and van der Waals interactions with one or more of the above residues. The default Discovery Studio 2.1 definition for H-bonds was used with H-bonds distances reported here between 3.0–3.1 Å (Accelrys, San Diego, CA).

**Figure 5:**
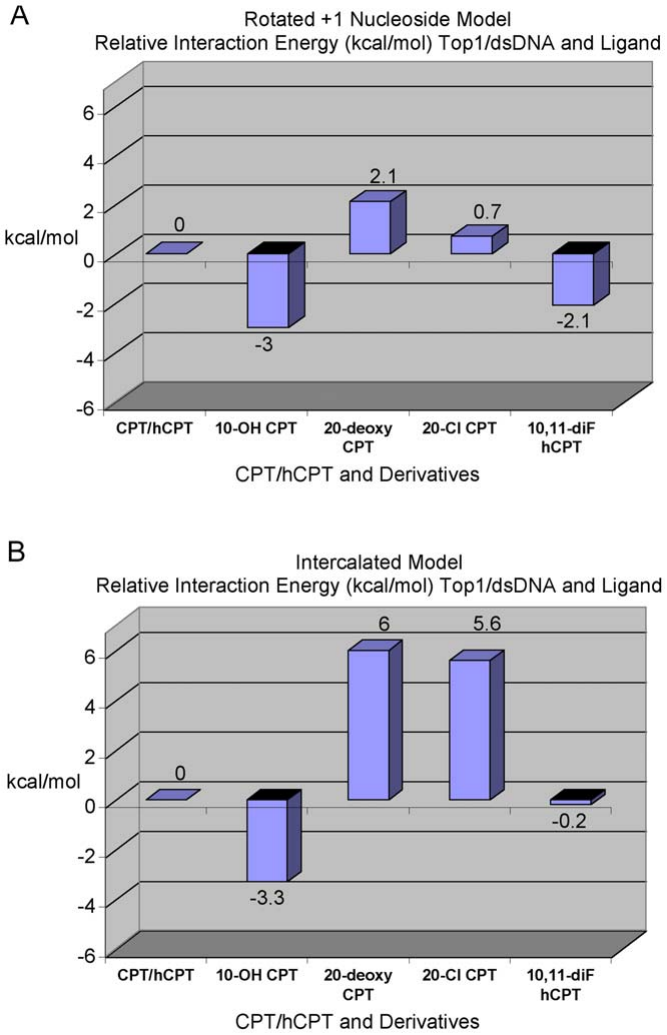
Relative interaction energy in kcal/mol between CPT/hCPT-derivatives and the Top1/dsDNA active-site. The interaction energy scores for the derivatives of CPT and hCPT were subtracted from the score of the respective parent inhibitor with the resulting difference values plotted on the graph. The CPT and hCPT interaction energy scores were set to zero. A negative kcal/mol score indicates that a derivative bound tighter than the parent inhibitor, while a positive kcal/mol score indicates that it bound weaker than the parent inhibitor. A) Interaction energy values for CPT, hCPT and derivatives when bound in the Rotated +1 Nucleoside model Top1/dsDNA active-site. B) Interaction energy values for CPT, hCPT and derivatives bound in the Intercalated model Top1/dsDNA active-site.

In order to evaluate the orientation of CPT and derivatives in the two models, and compare them to the reported *in vitro* activities for those same compounds (Figure 1 and Table 1), the interaction energy scores between CPT and derivatives and the active-sites of the Rotated +1 Nucleoside model (Fig. 2 and 4A) and the Intercalated model (Fig. 3 and 4B) were calculated (Fig. 5). For both models, the CPT interaction energy score (Rotated +1 Nucleoside model: vdW -48.6, E -4.67; Intercalated model: vdW -69.22, E -5.9) was subtracted from that for 10-OH CPT, 20-deoxy CPT, and 20-Cl CPT, while the hCPT interaction energy score (Rotated +1 Nucleoside model: vdW -49.33, E -1.92; Intercalated model: vdW -68.63, E -5.54) was subtracted from that for 10,11-diF hCPT. The resulting difference values for 10-OH CPT, 20-deoxy CPT, 20-Cl CPT, and 10,11-diF hCPT in the two models were then graphed to visualize whether an inhibitor bound either stronger (negative kcal/mol), or weaker (positive kcal/mol), than either CPT, or hCPT, which were set to zero (Fig. 5). For each of the following ligands the relative increase/decrease in the van der Waals (vdW) and electrostatic energy (E) is indicated. It has been reported that 10-OH CPT and 10,11-diF hCPT were 3.5-fold and 12-fold more potent *in vitro* than CPT and hCPT, respectively (Table 1) [15], [16]. It was also reported that the racemic 20-deoxy CPT had 3%, and racemic 20-Cl CPT had 29%, of the inhibition of CPT (Table 1) [20].

### Ligand scores for Rotated +1 Nucleoside model

In the Rotated +1 Nucleoside model, the 10-OH CPT value was -3.23 kcal/mol (vdW -2.16, E -1.07) stronger than CPT, and the 10,11-diF hCPT value was -2.03 kcal/mol (vdW -0.16, E -1.87) (Fig. 5A) stronger than hCPT due to either the A-ring 10-OH, or 10,11 diF, interacting with Asn352 (Fig. 2B and 4A). These additional interactions explained why 10-OH CPT and 10,11-diF hCPT were more potent *in vitro* than CPT and hCPT, respectively (Table 1) [15], [16]. The 20-deoxy CPT value was 1.81 kcal/mol (vdW 1.5, E 0.31) weaker than CPT (Fig. 5A) due to the loss of the 20-OH interaction with the Top1/dsDNA active-site (Fig. 5A). This is consistent with the racemic 20-deoxy CPT having low *in vitro* activity (Table 1) [20], with the 1.81 kcal/mol value (vdW 1.5, E 0.31) representing a loss of 6% of the total electrostatic interaction energy for CPT. Interestingly, the 20-Cl CPT value was only 0.49 kcal/mol (vdW 0.2, E 0.29) weaker than CPT (Fig. 5A). This result for 20-Cl CPT is consistent with the lack of a H-bond between the E-ring 20-Cl and the -1 scissile strand thymidine carbonyl oxygen (see E-ring 20-OH hydrogen in Fig. 2B and 4A). However, the 20-Cl CPT is significantly more potent *in vitro* than 20-deoxy CPT (Table 1), indicating that the 20-Cl is contributing to interactions between 20-Cl CPT and Top1/dsDNA through electrostatic interactions with Arg364 and Lys532. In the Rotated +1 Nucleoside model the Arg364 side-chain nitrogens make H-bonds to Asp533 and the -1 non-scissile strand cytosine and the +1 non-scissile strand adenine, and electrostatic interactions with the E-ring 20-OH (Fig. 4A). These interactions by Arg364 would contribute to the stabilization of the Top1/dsDNA/inhibitor complex (Fig. 2 and 4A). When Arg364 was mutated to His, the mutant Top1 became resistant to CPT and derivatives [23]. The shorter His side chain can neither make H-bonds to Asp533 and the -1/+1 non-scissile strand bases, nor electrostatic interactions with the E-ring 20-OH (data not shown). These *in silico* results support the binding orientation for CPT, hCPT, and derivatives in the Rotated +1 Nucleoside model (Fig. 2). However, it is important to note that while the 10,11 diF hCPT *in vitro* inhibition (Table 1, 12-fold) follows the respective Rotated +1 Nucleoside model difference value of -2.03 kcal/mol (Fig. 5A), the two values do not correlate in magnitude with the corresponding values for 10-OH CPT (Table 1, 3.5-fold; and Fig. 5A, -3.23). Fluorinated compounds have increased hydrophobicity [24], which *in vitro* likely results in 10,11-diF hCPT partitioning more efficiently out of the solvent phase and into the Top1/dsDNA binding cavity than does 10-OH CPT which is more hydrophilic due to the A-ring 10-OH. However, the interaction energy calculations in this study did not take that into account. Consistent with the reported 12-fold inhibition enhancement for 10,11-diF hCPT (Table 1), an inhibitor of glycogen phosphorylase, in which two hydroxyls were replaced with fluorines, was 10-fold more potent than the non-fluorinated inhibitor [25]. And while the addition of a hydroxyl to an inhibitor can be used to probe the active-site of an enzyme for a H-bond donor/acceptor, the substitution of a hydroxyl on an inhibitor with fluorine can be used to probe the active-site of an enzyme for the presence of just a H-bond donor [24], [25]. The *in vitro* data in Table 1 indicates that 10-OH CPT, 10,11-diF hCPT, and 20-Cl CPT interact with H-bond donors in the Top1 active-site. The Rotated +1 Nucleoside model supports this conclusion since Asn352 can donate H-bond/electrostatic interactions to 10-OH CPT and 10,11-diF hCPT (Fig. 2B and 4A), and Arg364 and Lys532 which are H-bond donors can make electrostatic interactions with 20-Cl CPT at ∼4 Å (data not shown).

### Ligand scores for Intercalated model

In the Intercalated model, the 10-OH CPT value of -3.31 kcal/mol (vdW -2.06, E -1.25) (Fig. 5B) was stronger than CPT due to the 10-OH CPT A-ring 10-OH making a H-bond to Glu356 (Fig. 3B and 4B). In the Staker et al. structure based Top1/suicide-DNA/topotecan model, the Glu356 side-chain oxygen was 3.8 Å from the topotecan A-ring 10-OH with a reported B-factor of 48 [18]. The topotecan A-ring 9-CH_2_-N-(CH_3_)_2_ may have disrupted the interaction between Glu356 and the adjacent A-ring 10-OH. In contrast, the 10,11-diF hCPT value of -0.2 kcal/mol (vdW -0.34, E 0.13) was only slightly stronger than hCPT (Fig. 5B), due to an electrostatic clash between the A-ring 10,11-diF and the Glu356 side-chain oxygens (note positive E value in the above score). These *in silico* results for 10,11-diF hCPT appear inconsistent with the reported *in vitro* 12-fold increase in inhibition (Table 1) [15]. As pointed out above, fluorine has been used to probe the active-site of enzymes for H-bond donors, however, the Glu356 side-chain oxygens can not donate a H-bond to fluorine. In the Intercalated model, the 20-deoxy CPT value was 6.03 kcal/mol (vdW 1.91, E 4.12) weaker than CPT with the electrostatic component representing a loss of 70% of the total electrostatic interaction energy for CPT (see above, Fig. 5B). The decrease in electrostatics for 20-deoxy CPT can be explained by the lack of a H-bond between the 20-deoxy CPT E-ring and Asp533; the racemic 20-deoxy CPT had low *in vitro* activity (Table 1) [20]. The 20-Cl CPT value of 5.64 kcal/mol (vdW 0.74, E 4.9) was also significantly weaker than CPT due again to the lack of a H-bond between the 20-Cl CPT E-ring and the Asp533 side-chain oxygen (see 20-OH group of 10-OH CPT in Fig. 3B and 4B). In the Intercalated model, the CPT 20-OH H-bond to Asp533 is the dominant electrostatic interaction between the inhibitor and the Top1/dsDNA active-site. However, in the Intercalated model the 20-Cl makes an electrostatic clash with Asp533 (see 20-OH of 10-OH CPT in Fig. 3B and 4B). The *in silico* results for 10,11-diF hCPT and 20-Cl CPT in the Intercalated model (Fig. 5B) do not follow the trend for the known *in vitro* inhibition by these CPT/hCPT derivatives (Table 1). It is important to note that these *in silico* results allow for further testing of both models. For the Intercalated model to be biologically relevant (Fig. 3 and 4B) mutation of Top1 Glu356 to Ala should result in a Top1 mutant that could not distinguish between CPT and 10-OH CPT due to the lack of a H-bond between an Ala356 and the A-ring 10-OH. In contrast, for the Rotated +1 Nucleoside model, the same Top1 Glu356Ala mutant should still be inhibited ∼3.5-fold more by 10-OH CPT, than by CPT, because in the Rotated +1 Nucleoside model the 10-OH CPT A-ring does not interact with Glu356 (Fig. 2B).

For this study, interactions between Top1 residues Glu356 and Asp533 and CPT derivatives were critical in showing that the Intercalated model did not support the structure-activity-relationships for chlorinated and fluorinated derivatives of CPT and hCPT, respectively (Table 1 and Fig. 5B). The results for the Intercalated model suggest that the use of suicide-DNA, and high inhibitor-to-enzyme ratios (∼34∶1) to generate Top1/suicide-DNA/inhibitor ternary complexes resulted in non-biologically relevant binding modes for CPT and derivatives [18], [19]. This is supported by the fact that the Top1/suicide-DNA/topotecan active-site was found to contain both the lactone (63%) and carboxylate (37%) forms of topotecan (the same ratio was also found for topotecan in mother liquor alone) [18]. The Top1/suicide-DNA/CPT active-site was also found to contain both the lactone and carboxylate forms of CPT, for which the percentages were so close they could not be differentiated [19]. In contrast, Top1 and dsDNA oligonucleotides were reported *in vitro* to preferentially stabilize the active lactone form of CPT (97.1%) versus the inactive carboxylate form (2.9%) [15]. The carboxylate form of hCPT, which can not reverse to the lactone form, has been shown to have no detectable inhibitory activity *in vitro* [15]. It can be argued that the Staker et al. Top1/suicide-DNA active-site could not distinguish between active and inactive forms of CPT and derivatives because the +1 nucleoside was not rotated out of the helix to open a biologically important inhibitor binding cavity (Fig. 2) [15]. The Rotated +1 Nucleoside model H-bond/electrostatic interactions between Arg364 and -1 non-scissile strand A and +1 non-scissile strand C, Arg488 and +1 scissile strand G, Lys532 and -1 scissile strand T, and Arg590 and +1 scissile strand G (Fig. 2B and 4A) are consistent with CPT stabilized Top1/dsDNA covalent-complexes occurring preferentially at sequences containing a -1 scissile strand T/+1 scissile strand G and followed by other combinations of -1 pyrimidines and +1 purines/pyrimidines [26].

Here it was also found that the lactone forms of CPT and derivatives made direct H-bonds to the Top1/dsDNA active-site in both the Rotated +1 Nucleoside and Intercalated models which is consistent with: 1) a previous report for a solvated Rotated +1 Nucleoside model [15]; 2) for the X-ray crystallography based Intercalated model [18]; and 3) molecular dynamic simulations of the topotecan lactone in the solvated Top1/dsDNA active-site by Mancini et al. [27].

The probing of the Top1 active-site with CPT derivatives containing chlorine and fluorine substitutions for hydroxyls revealed that 20-Cl CPT was able to make an electrostatic interaction with the Top1/dsDNA active-site, and 10,11-diF hCPT interacted with active-site H-bond donors near the A-ring. Only the binding of these inhibitors in the Rotated +1 Nucleoside model allows for 20-Cl CPT to maintain electrostatic interactions with Top1/dsDNA via Arg364 and Lys532, and for Asn352 to interact with fluorines on 10,11-diF hCPT (see 10-OH CPT in Fig. 2B and 4A). Together these findings further define the orientation of CPT and derivatives in the Top1 active-site, and allow for the rational design of CPT derivatives that make additional interactions with the Top1 active-site residues, including Asn430, Pro431, Leu721, and Lys751. This approach, in combination with the rational design of CPT derivatives which avoid cancer cell drug resistance mechanisms [9], [10], has the potential to result in more efficacious CPT derivatives for the treatment of drug resistant cancers and cancers that are currently not treated with CPT derivatives.

## Methods

### Structure based Top1/dsDNA Rotated +1 Nucleoside model

All Top1/dsDNA and inhibitor modifications, minimizations, and calculations, were carried out using Discovery Studio 2.0 with conditions and parameters that were optimized to mimic general *in vitro* conditions for direct interactions between ligands and an enzyme active site (Accelrys, San Diego, CA). 1) In the Redinbo et al. 1A31.pdb file [11], all waters were deleted, and all 5-iodo-2′-uridines in the DNA were replaced with thymidines. In the Staker et al. 1K4T.pdb file [18], topotecan (lactone and carboxylate forms), mercury, PEG, and all waters were deleted. 2) All incomplete Top1 side-chains were built out in the 1A31.pdb and 1K4T.pdb structures. 3) The 1A31.pdb Top1/suicide-DNA structure was superimposed with the 1K4T.pdb Top1/suicide-DNA/topotecan structure using selected atoms in the respective enzymes and bound dsDNAs. The aligned non-scissile strand nucleotides 115–122, and scissile strand nucleotides 1–10 in the 1A31.pdb and 1K4T.pdb structures had an RMSD of 0.598 Å. The 1A31.pdb Top1 protein was then deleted. 4) The intercalated topotecan in the 1K4T.pdb structure displaced the non-scissile strand nucleotides (101–114) and scissile strand nucleoside 11 and nucleotides 12–22, and so these portions of the DNA in the 1K4T.pdb structure were deleted leaving the corresponding DNA from the 1A31.pdb structure. The 1A31.pdb non-scissile strand bases 115–122 and scissile strand bases 1–10 in the DNA, that were aligned to the corresponding 1K4T.pdb bases (RMSD, 0.598 Å), were also deleted. The 1A31.pdb scissile strand nucleoside 11 free 5′ sulfur (S5′) was replaced with the native oxygen and the thymine base was replaced with a guanine (the 1K4T.pdb scissile strand base 11 is a guanine), and the base pairing non-scissile strand adenine in the 1A31.pdb DNA was replaced with a cytosine to allow for proper base pairing to the scissile strand guanine 11. Note: in the RCSB Protein Data Bank current “remediated” version of the 1A31.pdb entry, the scissile strand nucleoside 11 S5′ is incorrectly labeled as an O5′. 5) The backbone atoms of nucleotides 114/115 in the remaining non-scissile strand DNA (nucleotides 101–114 from 1A31.pdb and 115–122 from 1K4T.pdb) were bonded and the entire Top1/dsDNA covalent-complex was typed with CFF, the bond orders for the atoms in the tyrosyl-phosphate bond were corrected as needed, and then minimized with an Implicit Solvent Model using Implicit Distance-Dependent Dielectrics and no restraints using first Steepest Descent and then Conjugate Gradient algorithms to a Final Convergence of 0.1. 6) The +1 nucleoside was rotated, around the backbone oxygen-phosphorus bond, out of the helix until the guanine base and deoxyribose 5′OH made a network of H-bonds/electrostatic interactions with Arg488, Asp533, and Arg590 as previously reported [15]. 7) CPT was docked into the Top1/dsDNA active-site such that it made the same network of H-bond/electrostatic interactions with Top1 and dsDNA as previously reported [15]. CPT was then modified into the following derivatives: 20-deoxy CPT, 20-Cl CPT, hCPT and 10,11 diF-hCPT. Inhibitors were first typed with CFF and then the C-ring C15 and C16 atom types in hCPT and derivatives, and C10 and C11 atoms in CPT and derivatives, were manually corrected from cpb (bridge carbon biphenyl functional group) to cp (Sp2 aromatic carbon in either five- or six-member ring). Inhibitors were then minimized with an Implicit Solvent Model using Implicit Distance-Dependent Dielectrics and no restraints using Steepest Descent and then Conjugate Gradient algorithms to a Final Convergence of 0.1. 8) Next the entire Top1/dsDNA/inhibitor covalent-complex was typed with CFF (again, inhibitor C-ring C15 and C16 atom types in hCPT and derivatives, and C10 and C11 atoms in CPT and derivatives, were corrected from cpb to cp), and then minimized with an Implicit Solvent Model (implicit distance-dependent dielectrics, dielectric constant of 3), Electrostatics spherical cutoff, Nonbond List Radius of 14 (nonbond higher cutoff distance 12, nonbond lower cutoff distance 10), and no restraints using Steepest Descent and then Conjugate Gradient algorithms until either a RMS Gradient of 0.1, or Energy Change of 0.0, was reached.

### Structure based Top1/dsDNA Intercalated model

All modifications and minimizations were carried out using Discovery Studio 2.0 (Accelrys, San Diego, CA). 1) In the Staker et al. 1K4T.pdb [18] the topotecan carboxylate, mercury, PEG, and all waters were deleted. 2) All incomplete Top1 side-chains were built out and the scissile strand +1 nucleoside 5′ sulfur (S5′) was changed to the native oxygen. 3) The topotecan A-ring 9-N(CH3)_2_ was replaced with a hydrogen to give 10-OH CPT, and then the A-ring 10-OH was replaced with a hydrogen to give CPT. CPT was then modified into the following derivatives: 20-deoxy CPT, 20-Cl CPT, hCPT and 9,10 diF-hCPT. Inhibitors were first typed with CFF and then the C-ring C15 and C16 atom types in hCPT and derivatives, and C10 and C11 atoms in CPT and derivatives, were manually corrected from cpb (bridge carbon biphenyl functional group) to cp (Sp2 aromatic carbon in either five- or six-member ring) and minimized with an Implicit Solvent Model using Implicit Distance-Dependent Dielectrics and no restraints using Steepest Descent and then Conjugate Gradient algorithms to a Final Convergence of 0.1. 4) Next the entire Top1/dsDNA/inhibitor covalent complex was typed with CFF (inhibitor C-ring atom types were corrected from cpb to cp), and then minimized with an Implicit Solvent Model (implicit distance-dependent dielectrics, dielectric constant of 3), Electrostatics spherical cutoff, Nonbond List Radius of 14 (nonbond higher cutoff distance 12, nonbond lower cutoff distance 10), and no restraints using the Steepest Descent and then Conjugate Gradient algorithms until either a RMS Gradient of 0.1, or Energy Change of 0.0, was reached.

### Interaction energy calculations

All calculations were carried out using Discovery Studio 2.0 (Accelrys, San Diego, CA).

The interaction energy scores between inhibitors and Top1/dsDNA were calculated using an Implicit Solvent Model (implicit distance-dependent dielectrics, dielectric constant of 3) and a Nonbond List Radius of 14 (nonbond higher cutoff distance 12, nonbond lower cutoff distance 10). A dielectric constant of 3 was used to approximate the direct H-bonds between the ligands and the Top1/dsDNA active-site residues which excluded the presence of water (see Results and Discussion) [28], [29].
